# Supplementary material for: Resveratrol and Exercise Produce Recovered Ankle and Metatarsus Joint Movements after Penetrating Lesion in Hippocampus in Male Rats
Source: Brain Sci. 2024 Sep 27;14(10):980. doi: 10.3390/brainsci14100980 (PMC11506448; doi:10.3390/brainsci14100980)
Supplement: Supplementary file 1 [file brainsci-14-00980-s001.zip › brainsci-3191262-supplementary.pdf]

S1. **Timeline of the penetrating model.**

We averaged the vector Y components at each end of the normalized displacement curves for each group. Every step curve of an animal was compared to the curves of a different control animal, sequentially with each displacement curve of all animals in the control group. Thus, we calculated differences in the displacement curves between every animal and compared experimental versus control groups. We compared this pattern comparison analysis by using a locally designed MATLAB script.

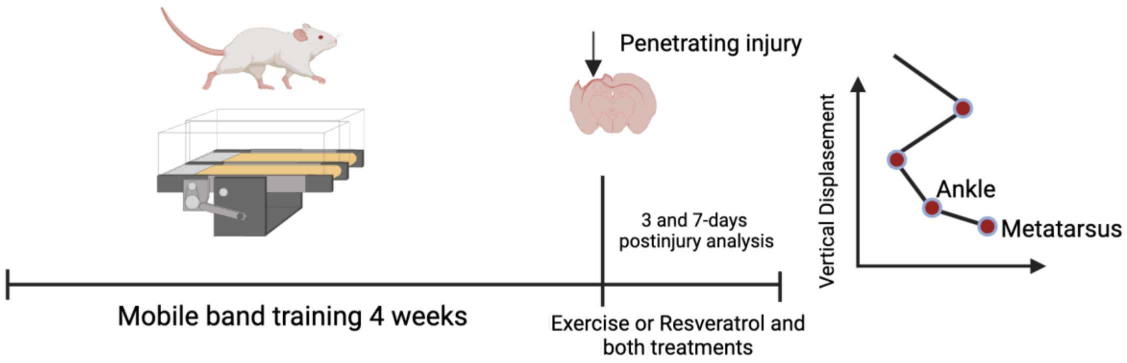

Figure S1. Timeline of the penetrating model. The rats walked for 4 weeks before and a week post-injury on the mobile band. Resveratrol treatment was administered for 7 days continuously after penetrating injury.

S2. **Vertical displacement in metatarsus on the third and seventh days after the lesion in male rats.**

To determine the changes in vertical displacement of adult male rats after a penetrating injury to the hippocampus, differences in the displacement of the metatarsal and ankle joints of the hindlimb were analyzed. It illustrates the vertical displacement of the metatarsus of the control group, the 3-dpi group, and the 7 dpi group. Asterisks above zero (*) show the points where the step cycle is different with statistical significance ($P \leq 0.05$). The annotation at the bottom of each graph shows the total percentage of the cycle that was statistically different.

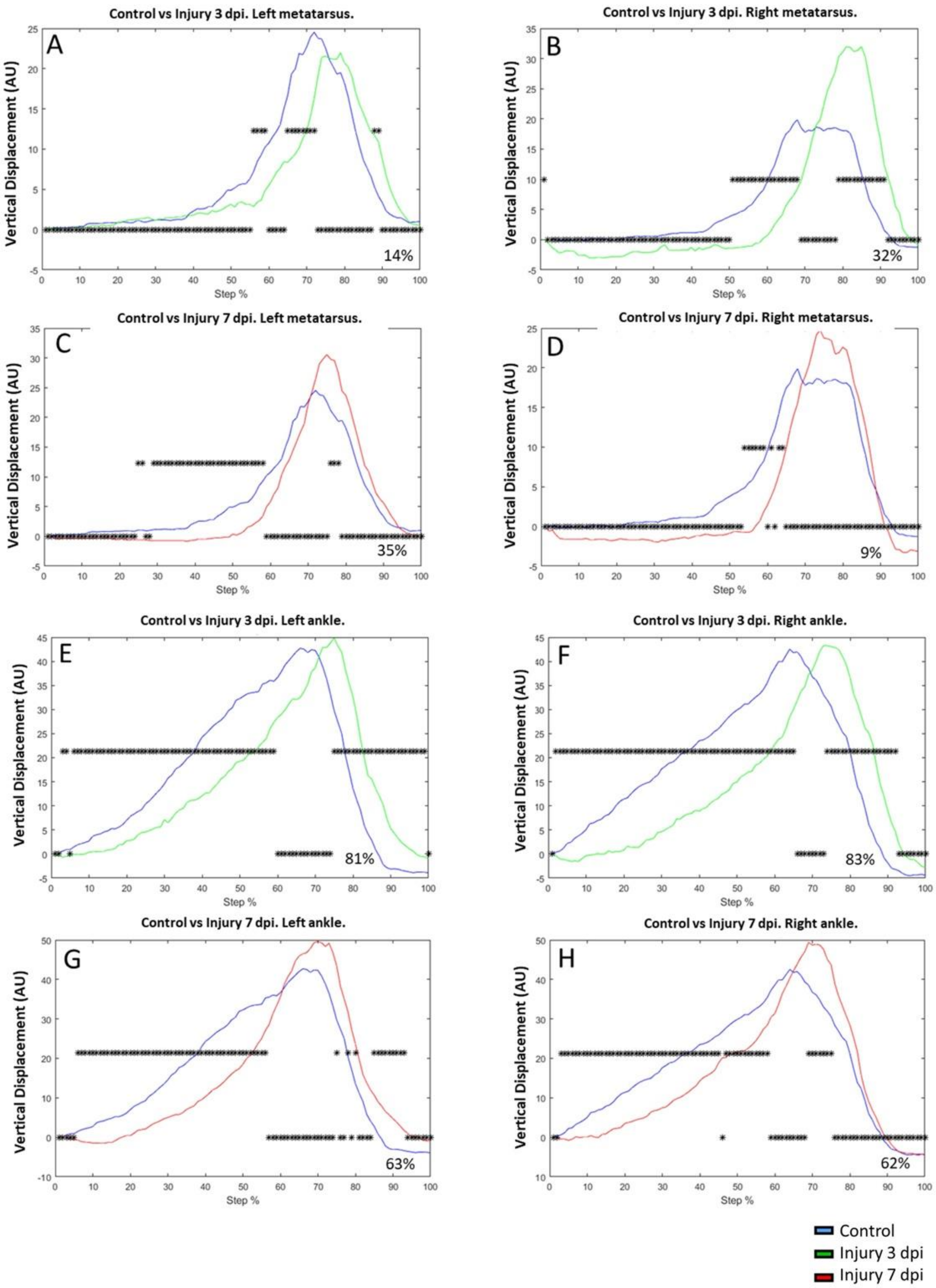

Figure S2. A-B, graphs illustrate the metatarsus vertical displacement (VD) of the control group (blue) versus the injury group at 3 days (3 dpi, green) in left and right metatarsus joints respectively. C – D, VD of the metatarsus control group (blue) versus the injury group at 7 days (7 dpi, red). The asterisks illustrate the bins with a statistical difference (*P ≤ 0.05). The percent of change is expressed below the graphs. E-F, graphs illustrate the ankle vertical displacement (VD) of the control group (blue) versus the injury group at 3 days after injury (3 dpi, green) in the left and right metatarsus joint respectively. G – H, VD of ankle joint control

group (blue) versus a group with injury at 7 days after injury (7 dpi, red). The asterisks illustrate the bins with a statistical difference (*$P \leq 0.05$). The percent of change is expressed below the graphs.

S3. **General changes in locomotion kinematics after penetrating injury and treatments.**

To have a broader overview of the changes in the kinematics of gait locomotion after penetrating injury and the effects of the different treatments (Exercise and Resveratrol), a data concentrate corresponding to the changes in the Vertical displacement (VD) and Horizontal displacement that occurred between the experimental groups on both the left and right sides (Figure 3). It is important to highlight the VD that had minimal significant differences (0 or 1% of the step cycle) whether on the left or right side. These comparisons between groups either at 3 or 7 dpi, which had minimal differences between them compared to the control group, tell us that the vertical or horizontal displacement was the same as the control despite the injury model.

				Vertical displacement		Horizontal displacement	
				Left	Right	Left	Right
Metatarsus	Injury 3 dpi vs Control			14	32	9	10
	Injury 7 dpi vs Control			35	9	0	1
	Injury 3 dpi vs Injury 7 dpi			0	6	29	13
	Ex 3 dpi vs Control			1	0	4	20
	Ex 7 dpi vs Control			1	0	45	36
	Ex 3 dpi vs Injury 3 dpi			12	23	24	46
	Ex 7dpi vs Injury 7 dpi			12	23	78	31
	Resv 3 dpi vs Control			26	21	14	21
	Resv 7 dpi vs Control			21	0	22	27
	Resv 3 dpi vs Injury 3 dpi			6	9	26	0
	Resv 7 dpi vs Injury 7 dpi			13	5	31	18
	Ex - Resv 3 dpi vs Control			14	0	10	0
	Ex - Resv 7 dpi vs Control			10	6	23	23
	Ex - Resv 3 dpi vs Injury 3 dpi			41	43	45	40
	Ex - Resv 7 dpi vs Injury 7 dpi			37	20	45	5
Ankle	Control vs Injury 3 dpi			81	83	69	62
	Control vs Injury 7 dpi			63	62	61	41
	Injury 3 dpi vs Injury 7 dpi			0	13	9	26
	Ex 3 dpi vs Control			30	11	24	46
	Ex 7 dpi vs Control			30	25	80	81
	Ex 3 dpi vs Injury 3 dpi			83	63	62	88
	Ex 7dpi vs Injury 7 dpi			82	67	92	69
	Resv 3 dpi vs Control			11	46	15	55
	Resv 7 dpi vs Control			18	12	27	26
	Resv 3 dpi vs Injury 3 dpi			35	49	27	42
	Resv 7 dpi vs Injury 7 dpi			64	52	82	59
	Ex - Resv 3 dpi vs Control			0	30	0	0
	Ex - Resv 7 dpi vs Control			26	9	27	6
	Ex - Resv 3 dpi vs Injury 3 dpi			72	79	68	68
	Ex - Resv 7 dpi vs Injury 7 dpi			54	56	62	59

Figure S3. Differences in Vertical and Horizontal displacement between the left and right side by group. The total differences of differences with statistical significance in the step cycle per joint in the VD between the left side and the right side of each experimental group are shown. A data concentrate corresponding to the changes in the Vertical displacement (VD) and Horizontal displacement that occurred between the experimental groups on both the left and right sides.
